# *Ciona* as a Simple Chordate Model for Heart Development and Regeneration

**DOI:** 10.3390/jcdd3030025

**Published:** 2016-08-09

**Authors:** Heather Evans Anderson, Lionel Christiaen

**Affiliations:** 1 Department of Biology, Winthrop University, Rock Hill, SC 29732, USA; 2 Department of Biology, New York University, New York, NY 10003, USA; lc121@nyu.edu

**Keywords:** *Ciona*, cardiac development, cardiac cell specification, cardiac regeneration

## Abstract

Cardiac cell specification and the genetic determinants that govern this process are highly conserved among Chordates. Recent studies have established the importance of evolutionarily-conserved mechanisms in the study of congenital heart defects and disease, as well as cardiac regeneration. As a basal Chordate, the *Ciona* model system presents a simple scaffold that recapitulates the basic blueprint of cardiac development in Chordates. Here we will focus on the development and cellular structure of the heart of the ascidian *Ciona* as compared to other Chordates, principally vertebrates. Comparison of the *Ciona* model system to heart development in other Chordates presents great potential for dissecting the genetic mechanisms that underlie congenital heart defects and disease at the cellular level and might provide additional insight into potential pathways for therapeutic cardiac regeneration.

## 1. Introduction

The animal species comprising the phylum Chordata display very diverse cardiac morphologies and physiology. Cardiac features are highly-adaptive such that diffusely contractile vessels in cephalochordates, simple peristaltic compartments in Tunicates, and multi-chambered pumps with separate high and low pressure circulation in amniotes reflect the evolution and divergence of Chordate species. Yet, across this diversity lies a deeply conserved network of genetic determinants for cardiac cell specification and patterning. Classical examples of key cardiac developmental control genes include orthologs of the transcription-factor-coding genes *Nkx2.5, Gata4/5/6, Hand1/2*, and *Tbx1* that are highly conserved across Chordate species and beyond. These key cardiac gene determinants are thought to form a regulatory kernel, or conserved core subnetwork, that determines cardiac cell specification and differentiation, resulting in conserved cardiac cell types and cellular organization of Chordate hearts [1,2]. Notably, the mechanism of cardiac cell type specification is not only conserved among Chordates, but also determines circulatory pump formation in *Drosophila* [3], which demonstrates the deep evolutionary origins of this kernel, whereas its widespread conservation presumably results from continuous selective pressure, highlighting its importance for cardiac development in bilateral animals.

The complex events that span from genetic regulation to cellular specification present a puzzle that must be solved in order to fully understand the underlying blueprint of Chordate heart development [4,5]. However, if the cellular and molecular underpinnings of congenital heart defects/disease (CHD) can be studied in simpler Chordates independently of their complex morphogenetic outcomes in higher vertebrates, then solving the riddle of heart development becomes more plausible. Thus, examining the conserved regulatory genetic linkages that lead to cardiac cell specification and differentiation, as well as the resulting cellular outcomes in the hearts of simple Chordates, presents an opportunity to gain insights into the deeply conserved mechanisms of heart development. Comparison of cardiac cell lineages and regulatory mechanisms of heart development across Chordate species elicits many questions. The first of which being, how can the regulatory kernel of genetic programming be so highly conserved and yet produce such great divergence of heart morphologies within the super-phylum Chordata? Furthermore, if cardiac cell specification during heart development and the resulting cardiac cell types that form the heart are also highly conserved in Chordates, then how do some species retain the ability to continuously produce new cardiac cells to replace and regenerate their hearts whereas other species do not?

Here we will focus on the development and cellular structure of the heart of the ascidian *Ciona* as compared to other Chordates, principally vertebrates. Examining cardiac fate-specification programs and conserved regulatory states of cardiac progenitor cells during *Ciona* heart development, as compared to vertebrates, presents an opportunity to examine the basic building blocks of the Chordate heart program in a simple model system. Moreover, studies in a simpler model system such as *Ciona*, which can naturally regenerate cardiac tissue, unlike adult mammals, with a simpler complement of cardiac genes compared to zebrafish, might shed light into the basic cellular and molecular requirements to reactivate the ancestral aspects of the cardiac program in a regenerative context. Thus, a comparison of the *Ciona* model system to heart development in other Chordates presents great potential for dissecting the genetic mechanisms that underlie congenital heart defects and disease at the cellular level, as well as provides insight into potential pathways for therapeutic cardiac regeneration.

## 2. Chordate Circulatory Systems: From Divergent Anatomies to Conserved Cytology

### 2.1. Divergent Cardiac Anatomy and Histology

**Figure 1:**
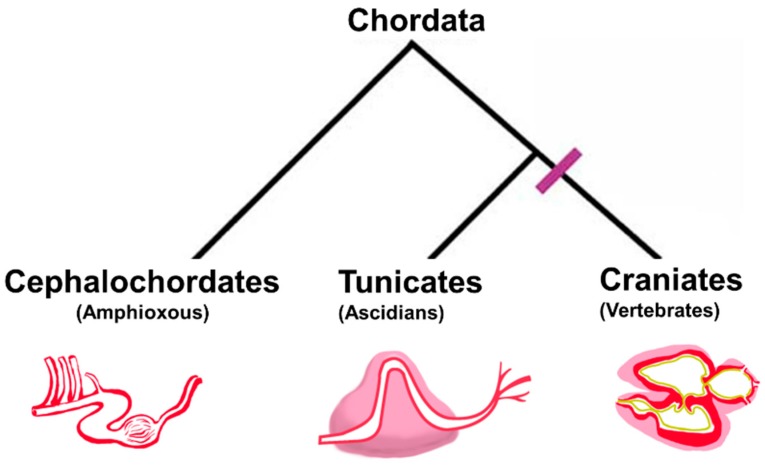
Phylogenetic relationship and general heart structure of Chordate subphyla. Cephalochordates have a series of four peristaltic vessels that serve as a pump. Tunicates have a single-chamber peristaltic pump comprised of a single layer of myocardium (**red**) surrounded by a pericardium (**pink**). Vertebrates have at least a two-chambered myocardium comprised of layered cardiac myocytes (**red**), an endocardium (**yellow**), valves that separate distinct inflow and outflow chambers and a surrounding pericardium (**pink**).

Within the super-phylum Chordata, Cephalochordates, Tunicates, and Craniates are phyla that share common embryonic features including a dorsal nerve cord, a notochord, and a post-anal tail [6]. Ascidians are a class of Tunicates and are primarily soft-bodied filter-feeders that are sessile, which lends to their common name of “sea squirts”. Cephalochordates, commonly known as lancelet or amphioxus, retain Chordate features as adults, and they were previously thought to be more closely related to Craniates. However, phylogenomic studies indicate that Tunicates are the true sister clade to the Craniates, which includes vertebrates [7,8]. Unlike shared embryonic features, the circulatory systems of Chordates vary widely and the final morphogenetic outcomes of heart development in Chordates are highly divergent. The general structure of Chordate hearts and the phylogenetic relationships are represented in Figure 1. Cephalochordates use non-striated muscle cells to power a series of four peristaltic vessels that circulate blood. Within Tunicates, the cardiac anatomy in Ascidians consists of a single-compartment peristaltic pump comprised of a mono-layer of striated muscle myocardium enclosed in a pericardial sac [9]. By contrast, Craniates have many well characterized model systems of heart development and anatomy, which generally display a striated, multi-layered, and trabeculated muscle cell myocardium that is divided into multiple chambers by valves with dedicated inflow and outflow, an endocardium, and enclosed by a pericardium.

Notably, the myocardium of both Cephalochordates and Tunicates, which consist of a monolayer of cardiac myoepithelial cells that lack an endocardium, is also characteristic of other invertebrate blood pumps, such as *Drosophila* [10]. Interestingly, other invertebrates outside of the super-phylum Chordata also display multi-layered myocardium despite lacking endo- and epi-cardial layers. Species of blue crab and oyster have trabeculae [11]. Whereas cephalopods, which have the most complex circulatory systems as invertebrates, display a closed circulatory system with a heart that has a thickened myocardium that is capable of producing powerful contractions [10,12]. Evidence of thickened myocardium in invertebrates outside the super-phylum Chordata suggests that there are other mechanisms besides endocardial and epicardial signaling that can result in myocardium layering. Thus, comparison of the mechanisms of heart development between all Chordates provides important insights into evolutionary advances; and, there is still much to learn about the genetic and morphometric regulation of heart development across all phyla. However, the fate specification programs and resulting cardiac cell types are most conserved among Chordates. As simple Chordates within the phylum Tunicata, the ascidian species of *Ciona* provide an excellent model system in which to examine the basic mechanisms of heart development.

Within Tunicates, species of the *Ciona* genus are among the most studied ascidians and there are multiple species of *Ciona* that have been characterized, whereas *Ciona savignyi* and *Ciona intestinalis* type A provided the first two whole-genome sequences for invaluable comparative analyses [13,14,15,16,17], recent evidence suggests that *Ciona intestinalis* type A and type B are in fact two separate species. *Ciona intestinalis sensu stricto*, formerly known as *Ciona intestinalis* type B, is found in the North Atlantic Ocean and *Ciona robusta*, formerly known as type A, is found in the Pacific Ocean [18]. In this review, we collectively refer to all of these species as *Ciona* since they all share the conserved genes, cardiac cell types, and heart structure.

### 2.2. Cellular Structure of Ciona Heart

Heart formation in *Ciona* occurs throughout embryonic, larval, juvenile, and adult stages. Larvae develop, hatch, and swim 12 to 18 h post-fertilization, and then attach to a substrate and metamorphose into sessile juveniles with beating hearts within 3–5 days. Juveniles continue to develop into young adults by 10 days post-fertilization, then grow isometrically as adults, mature within 1–3 months and die within 12–18 months [19,20,21,22]. There is a positive correlation between age and body length [23]. Since the embryonic and larval stages retain Chordate features and are more amenable to experimentation, most studies investigating *Ciona* heart development have focused on the characterization of early development up to the larval stage.

During embryogenesis, *Ciona* development is highly reproducible, with cell lineages, cell-cell contacts, and successive fate specification events being invariant and designated in part by intrinsic maternal determinants and well stereotyped cell-cell signaling events [24,25,26]. Cell lineage tracing has identified cardiac progenitor cells throughout embryonic development to the larval stage [27,28,29,30,31]. The genetic programs for early cardiac cell fate specification in *Ciona* share many of the same pathways that govern vertebrate heart development [32,33,34,35]. Cardiac progenitor cells specified during embryogenesis begin to differentiate during metamorphosis in order to form the beating heart [30,36]. Specifically, fate-restricted cardiac precursors arise in swimming larvae from multipotent cardiopharyngeal progenitors that also produce the atrial siphon and body wall muscles (a.k.a. pharyngeal muscles), in a manner analogous to that described in the mouse, where common progenitors of the second heart field cardiomyocytes and branchiomeric head muscles emerge from the primitive streak [37,38].

**Figure 2:**
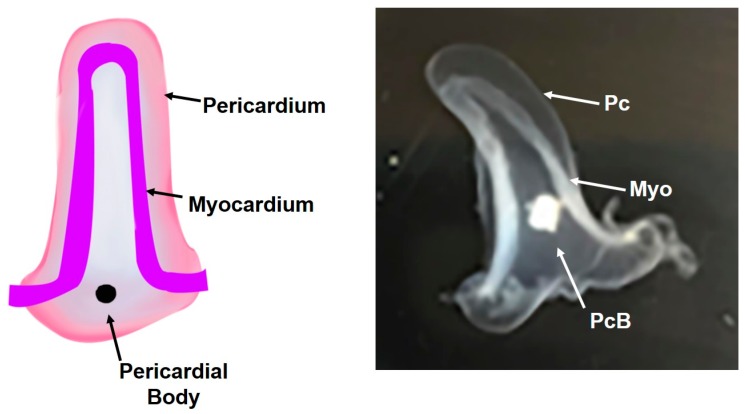
Adult Ciona Heart Structure. The heart primarily consists of the myocardium (Myo) and the pericardium (Pc). The pericardium encloses the myocardium. As the Ciona ages, a pericardial body (PcB) appears, which consists of decaying cardiac myocytes that are shed from the myocardium.

The main cell types that arise from cardiac progenitor cell differentiation include contractile cardiac myoepithelial cells and an epithelium of pericardial cells. It is not clear how the cardiac progenitor cells in *Ciona* larvae eventually produce the diverse cell types observed in the post-metamorphic heart. The newly-formed heart can be seen beating 3–5 days post-fertilization in juvenile *Ciona* (see Video S1). The developmental stages and organ formation up to the juvenile stage of *Ciona* was reviewed previously [39]. During post-metamorphic development, the *Ciona* heart is initially round-shaped, then becomes V-shaped about 21 days post-fertilization [39]. There is very little information about the adult *Ciona* heart in the current literature; however, the most comprehensive resource is a report by R.H. Millar published in 1953 [20]. The adult *Ciona* heart grows in proportion to the body size of the animal and is a tubular, looped structure that consists of a myocardium with attachments to a pericardium that encloses the myocardium in a fluid-filled cavity (Figure 2). The *Ciona* circulation is open. The peristaltic contractions of the *Ciona* heart are rhythmic and directional, even though the direction of the contraction can be reversed (see Video S2).

**Figure 3:**
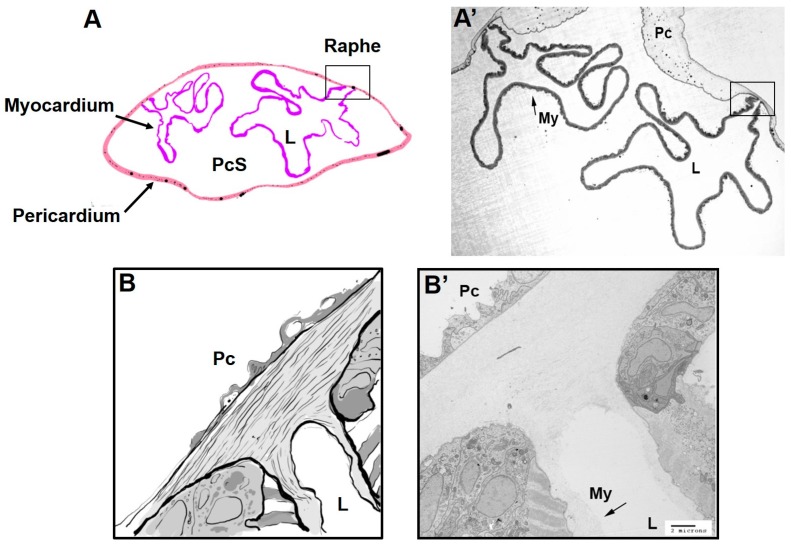
Relationship between the pericardium and the myocardium. (**A**) Cartoon representation of transverse section of adult *Ciona* heart imaged by TEM in **A’**. The myocardial tube forms a lumen (L) that is surrounded by the pericardial space (PcS). The pericardium (Pc) joins the myocardium (My) at the raphe (boxed region in **A** and **A’**); and (**B**) cartoon representation of raphe as imaged by TEM in **B’**. Between the cells of the outer pericardium and myocardium, the space is filled with an extracellular matrix that continues into the lumen of the myocardium (arrow, **B’**). Transitional cardiac myocytes exist where the inner layer of the pericardium transitions to the myocardium. Scale bar = 2 microns.

The cell types that make up the adult *Ciona* heart are cardiac myocytes; transitional myocardial cells; general and junctional pericardial cells; and cells of the undifferentiated line [9,20,40]. Transitional cardiac myocytes exist at the junction where the pericardium and myocardium meet (Figure 3). These cells are in various stages of differentiation wherein the formation of myofilaments can be observed towards the basal sarcolemma. The pericardium is comprised of a monolayer of non-contractile epithelial cells. The pericardium and myocardium are continuous at the raphe (Figure 3). An acellular extracellular matrix fills the space between the pericardiac and epicardiac walls and becomes more fibrous near the raphe. The cardiac raphe is a structural feature of the adult *Ciona* heart that occurs at the intersection of the myocardium and the pericardium (Figure 3). As the animal ages, a pericardial body appears from discarded and degenerating cells from the myocardium that resides within the pericardial space. The *Ciona* myocardium does not have endothelial cells that form an endocardium. However, the surface of cardiac myocytes that face the lumen of the heart is covered by a thin sheet of non-cellular matrix that is continuous with the blood vessels that extend away from the heart. Movat’s pentachrome staining of the *Ciona* heart shows that this extracellular matrix lining the heart is proteoglycan based. Further studies are necessary to confirm the identity and the role of this extracellular matrix.

**Figure 4:**
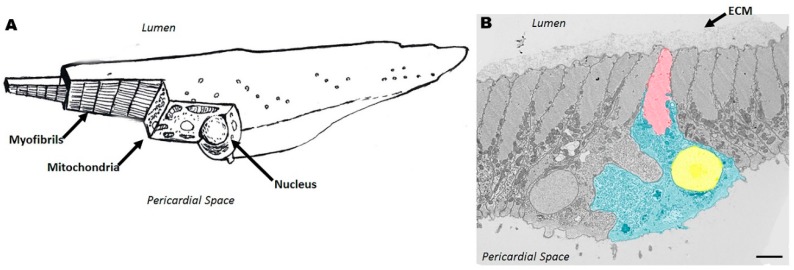
Cardiac Myocyte in the *Ciona* myocardium. (**A**) Schematic of a cardiac myocyte with sectioned areas to show intracellular organization; and (**B**) false-colored TEM of *Ciona* myocardium with highlighted cardiac myocyte (**red** = myofibrils; **yellow** = nucleus; **blue** = sarcoplasm). Unknown extracellular matrix is continuous on sarcolemma of cardiac myocytes facing lumen of heart. The nucleus is oriented towards the pericardial space. Scale bar = 2 microns.

Cardiac myocytes in *Ciona* are polarized cells that have nuclei oriented toward the apical pole facing the pericardium and myofilaments on the basal side facing the lumen of the heart (Figure 4). Myofilaments are striated and widely extend away from the central portion of the cell containing the nucleus. Binucleation occurs in ascidian cardiac myocytes, but the rate of occurrence has not been quantified. *Ciona* cardiac myocytes are in excess of 100 microns in length [20]. The myofilaments make an angle of 60 to 70° with the raphe, which runs parallel to the long axis of the heart [20]. This results in a slight spiral orientation to the myofilaments, which promotes directional contraction of the myocardium in the adult heart (see Video S2).

## 3. Development of the *Ciona* Heart and Parallels with the Vertebrates

Previous studies have shown deep conservation of specific regulatory networks that govern cardiac cell lineage specification [29,34]. The *Ciona* genome contains single copies of orthologous genes that govern cardiac development, including *Gata4/5/6*, *Nkx2.5*, and *Hand* gene families [41,42,43]. *Ciona intestinalis* type A/*C. robusta* has a small genome compared to vertebrates (115.2 Mb) [44]; and Tunicates appear to have diverged before whole genome duplications occurred [7]. Therefore, *Ciona* retained most of the basal Chordate program for cardiac development without the redundancy of the gene duplications seen in vertebrates.

**Figure 5:**
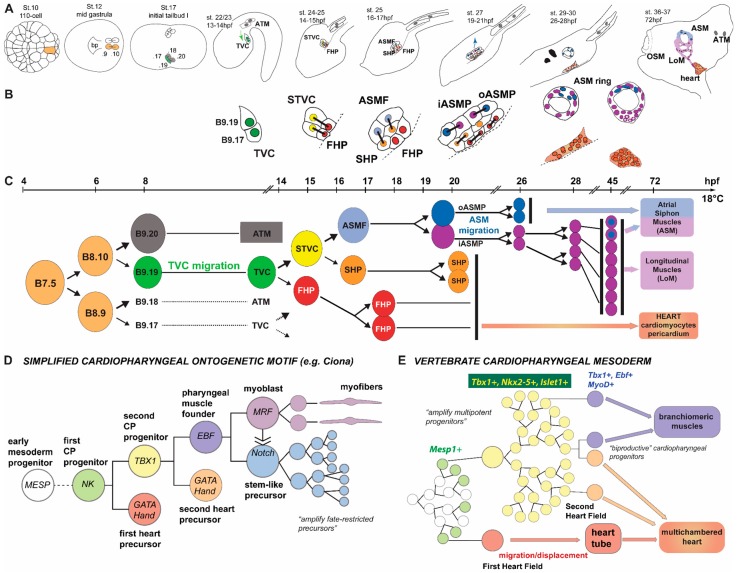
Summary of cardiac cell lineage specification in *Ciona*. (**A**) Schematic embryos, larvae, and juvenile showing the B7.5-derived cardiopharyngeal lineage, with approximate hours post-fertilization (hpf) and stages according to [59]. Numbers in the left second and third panels are simplified version of cell names according to Conklin (1905) nomenclature and corresponding the (**C**). Arrows indicate collective TVC (**green**) and ASMP (**blue**) migrations. TVC: trunk ventral cells; ATM: anterior tail muscles; STVC: second TVC; FHP: first heart precursor; SHP: second heart precursor; ASMF: atrial siphon muscle founder cells; iASMP: inner atrial siphon muscle precursor; oASMP: outer atrial siphon muscle precursor; LoM: longitudinal body wall muscles (note that they derive from the iASMPs); OSM: oral siphon muscles, derived from the A7.6 lineage [60]; bp: blastopore; and (**B**) close-up views of cardiopharyngeal lineage cells. Black bars link sister cells (as per the lineage shown in C). White asterisks in the ASM ring at ~72 hours post fertilization indicate iASMP-derived LoM precursors that reactivate Mrf and the body wall muscle differentiation program [31]. Note that the relative contributions of the FHP and SHP to the different parts of the differentiated heart (epicardium, myoepithelium, raphe, and pacemakers) remain elusive; (**C**) Lineage representation of cadiopharyngeal mesoderm development. Color codes correspond to those used in A and B. Only the progeny of one TVC for one side of the animal is shown, but this pattern is reiterated 4 times in each developing embryo, such that the *Ciona* equivalent of the first and second heart fields (see B) are composed of distantly-related FHP and SHP. For example, the left second heart field is composed of the anterior/leader and posterior/trailer SHPs, whose last common ancestor is the *Mesp*+ left B7.5 blastomere; (**D**) simplified cardiopharyngeal ontogenetic motif as seen in Ciona, illustrating that in ascidian embryos, cell fates are first restricted to a few progenitors, which are secondarily amplified (as seen for both the ASMP, and heart precursors); and (**E**) schematic representation of equivalent cardiopharyngeal ontogeny in vertebrates, where multipotent progenitors from larger populations in the early embryos, these morphogenetic fields are patterned by cell-cell signaling causing spatially-defined progressive fate restrictions such that not every single multipotent progenitor expresses its full potential. This may explain why a small fraction (~10%) of the bipotent cardiopharyngeal progenitors produce both skeletal head muscles and SFH-derived cardiomyocytes, whereas these populations are easily switched to one fate or another experimentally. The existence of such plastic populations of multipotent progenitors probably fostered the diversification of cardiopharyngeal structures in vertebrates.

There has been comprehensive mapping of the early heart lineage during ascidian embryogenesis [28,29,30,31,45]. A current summary of known cardiac cell lineage specification and the ontogenetic motif is illustrated in Figure 5. The entire *Ciona* heart is derived from only two bilateral blastomeres in the gastrulating embryo (St. 12), which express the conserved pre-cardiac specification factor *Mesp* [28,46]. Likewise, in vertebrates the transcription factor *Mesp1* is essential to mesoderm patterning and results in the formation of hematopoietic, skeletal muscle and cardiac cell lineages [37,47,48,49]. *Mesp*-expressing cells in *Ciona* divide once in early gastrula embryos to produce equivalent multipotent progenitors referred to as founder cells [50]. The founder cells divide again at the end of gastrulation (St. 22–23), this time asymmetrically, to generate two daughter cell lineages: the anterior tail muscle precursors (ATMs) and the trunk ventral cells (TVCs) [28]. All *Ciona* heart cells are derived from the TVCs, which are induced by FGF-mediated activation of the Ets1/2 transcription factor. This promotes the TVC-specific activation of conserved cardiac regulators including *Gata4/5/6*, *Nk4/Nkx2-5*, and *Hand* [28,51,52]. Notably, FGF also activates Ets1/2 transcription factors to initiate orthologous cardiac genes and cardiac cell specification in vertebrates [53]. In addition, similar to cardiac progenitor cells in vertebrate heart development, pairs of bilateral clusters of TVCs migrate towards the ventral midline where they meet to form a single cardiac progenitor pool in *Ciona* [46,51,52,54]. The bilateral fusion of cardiac progenitor cells to form a common progenitor pool is a critical step in vertebrate heart development. Alteration of fusion of cardiac progenitor cells results in *cardia bifida* phenotypes, whereby bilateral rudimentary hearts develop on either side of the ventral midline. Remarkably, perturbing gene function in the trunk epidermis can produce analogous failure of cardiac progenitors to merge at the midline in *Ciona* [55]. TVCs are multipotent cardiopharyngeal progenitors that will then divide asymmetrically and along the mediolateral axis to produce small median first heart precursors (FHPs) and large lateral second TVCs (STVCs) [30]. STVCs specifically activate the conserved transcription regulator *Tbx1/10* before dividing again asymmetrically and along the mediolateral axis to produce large lateral atrial siphon muscle founder cells (ASMFs) and small median second heart precursors (SHPs; [30,36]). All cells then divide along the anteroposterior axis before the lateral-most atrial siphon muscle precursors (ASMPs) begin to collectively migrate dorsally, towards the atrial siphon placode, where they form a conspicuous ring of ASM precursor cells [30]. Remarkably, ASMFs successively activate the myogenic regulators Ebf and Mrf before the birth of distinct ASMPs, which sets the stage for Notch-mediated lateral inhibition. This process distinguishes between *Mrf*- undifferentiated stem-cell-like muscle precursors and *Mrf*+ differentiating myoblasts [31]. Shortly before and during ASMP migration, all cardiopharyngeal cells activate *Islet*, the sole homolog of the classic vertebrate second heart field marker *Islet1* [56,57,58], which becomes restricted and more highly expressed in the migratory ASMPs [30,36].

These studies have not only provided novel insights into the evolutionary origins of the vertebrate first and second heart fields, but they also lend much insight into the origins of the branchiomeric muscles of the face, jaw and neck [38]. Briefly, branchiomeric muscles and cardiac progenitors share a common origin in the *Nkx2-5*+, *Tbx1*+, and *Isl1*+ anterior splanchnic/pharyngeal mesoderm, which derive from *Mesp1*+ mesoderm progenitors and has recently been referred to as the cardiopharyngeal field [38,58,61]. Retrospective clonal analyses in the mouse revealed the ontogenetic kinship between distinct head and neck muscles and specific derivatives of the second heart field [62,63]. These studies led to the proposal that the pattern observed in *Ciona* represents a simplified version of an ancestral/ancient cardiopharyngeal ontogenetic motif, whereby multipotent progenitors progress through transient regulatory states marked successively by *Mesp1*, *Nkx2-5*, *Tbx1*, and *Islet1* homologs and produce distinct first and second heart field progenitors, and pharyngeal muscles precursors and associated stem cells (Figure 5); [36,38].

More recent prospective clonal analyses, using state-of-the-art mouse genetic methods, further refined the clonal relationships between distinct cardiac and branchiomeric progenitors. These studies highlighted profound differences between early heart development in the mouse and in ascidians [37,49,64]. For instance, whereas previous analyses indicated that the first heart field (FHF) and second heart field (SHF) emerge from early common pan-cardiac progenitors [65], prospective clonal analyses using *Mesp1-rtTA* inducible Cre alleles revealed that *Mesp1* is activated independently in approximately 250 separate FHF and SHF progenitors that emerge at distinct time points in the primitive streak. These analyses also challenged the view that multipotent cardiovascular progenitors generate cardiomyocytes, endocardium and smooth muscles as all FHF progenitors appeared unipotent. By contrast, a fraction of the SHF progenitors produced either cardiomyocytes and smooth muscles, or cardiomyocytes and endocardial cells, or cardiomyocytes and branchiomeric skeletal muscles [37]. The latter represented ~10% of the total pool of cardiopharyngeal progenitors. Remarkably, this contrasts with the observations that every single one of the four cardiopharyngeal progenitors in ascidians expresses its full potential by generating both first and second heart precursors, and pharyngeal muscle progenitors [30,33,36]. Moreover, studies using explants and perturbations of FGF, BMP, and Wnt signaling in vertebrates demonstrated that cardiopharyngeal progenitors have the potential to generate either cardiomyocytes or skeletal muscles, depending on extrinsic conditions [58,61,66,67,68,69]. Finally, studies using mouse embryonic stem cells showed that forced expression of Mesp1 can promote either skeletal or cardiac muscle differentiation and even generate bipotential cardiopharyngeal progenitors depending upon the culture conditions [70,71]. These observations suggest that, whereas not every single cardiopharyngeal progenitor is actually “multi-productive” in early mouse embryos, there exist plastic populations of multipotent progenitors patterned by cell-cell signaling, which may have fostered the diversification of cardiac and pharyngeal structures during vertebrate evolution [38]. By contrast, the extreme simplification of ascidian cardiopharyngeal development led every single fate choice to be hard-wired, with very little modification in evolution [72], and each one of the four cardiopharyngeal progenitors expresses its full potential in every developing embryo. In terms of cardiopharyngeal clonal dynamics, some of the main differences between ascidians and vertebrates can, thus, be summarized as follows: In vertebrate embryos, multipotent progenitor population are produced and amplified before fate choices occur, whereas in ascidians fate choices occur early and stereotypically within small populations of cells followed by amplification of fate-restricted precursors populations (Figure 5D,E).

While the conserved gene regulatory networks that underlie early cardiac patterning and fate specification in *Ciona* are increasingly well characterized in embryos and swimming larvae (recently reviewed by [33,34,73]); the subsequent cell fate specification events, behaviors and gene activities are not characterized post-metamorphosis in *Ciona* juveniles or adults, and this remains an important area for future research.

## 4. *Ciona* as a Model for Basic Cellular and Molecular Aspects of Congenital Heart Defects

Many congenital heart defects (CHD) are compatible with life, amenable to surgery and, presumably, arise from complex morphogenetic defects that often impact the derivatives of the second heart field [74]. Whereas terminal cardiac organogenesis is mammalian-specific and without counterpart in simpler invertebrates, morphogenetic defects are often caused by genetic alterations of deeplyyconserved cardiac transcription regulators, which function in early embryonic progenitors. Therefore, it is plausible that understanding the function of conserved transcriptional regulators in the cardiopharyngeal progenitor cells of invertebrate Chordates, like *Ciona*, will provide novel insights into the immediate cellular and molecular consequences of mutations that cause CHD in humans. For example, *TBX1* haploinsufficiency resulting from 22q11.2 deletions is thought to cause cardiac and pharyngeal apparatus defects in the common cardiovelofacial/Di George syndrome [75], and Tbx1 mutations phenocopy 22q11-induced defects in mouse models [76,77,78], whereas the etiology of this syndrome is rather complex, there is mounting evidence that multiple defects arise from alteration of TBX1 function in cardiopharyngeal mesoderm progenitors of early embryos [79,80,81,82]. In this regard, the conserved expression of *Tbx1/10* homologs in the second cardiopharyngeal progenitors of ascidians might provide insights into conserved regulatory functions. For instance, *Tbx1/10* homologs act upstream of conserved myogenic regulatory factors, such as *MyoD*, as key determinants of pharyngeal myogenesis in both vertebrates and ascidians [36,83,84,85]. Studies using *Ciona* first identified a role for Collier/Olf/Ebf (COE) homologs in promoting pharyngeal myogenesis, by acting downstream of *Tbx1/10* and upstream of Mrf, and opposing cardiogenesis [30,31]. Notably, COE homologs have since been showed to regulate *MyoD* expression and myogenesis, including branchiomeric muscle formation, in *Xenopus* and chick embryos [86,87,88]. We propose that *Tbx1/10*, *Ebf* and *Mrf* homologs are part of an ancient transcriptional network for pharyngeal muscle specification [85], with a role in opposing cardiac development in the cardiopharyngeal lineage. Human *EBF* homologs may, thus, very well be genetic modifiers of the craniofacial phenotypes observed in 22q11.2 deletion syndrome patients.

Along this line, Tbx1 is thought to delay cardiac differentiation in the mammalian second heart field, allowing the progenitors to proliferate and produce the large amount of cells required to build the right ventricle and outflow tract, among the main SHF derivatives [81,82,89]. Certain defects observed in *Tbx1* mutants have been interpreted as failure of the SHF progenitors to proliferate. At the molecular level, Tbx1 interferes with the function of BMP-Smad signaling and the expression of Gata4/5/6 [90,91]. Remarkably, the latter function is conserved in *Ciona* where Tbx1/10 activity in the multipotent STVC progenitors contributes to delay the re-activation of *Gata4/5/6* in the second heart field [36]. This study also showed that Tbx1/10 could activate *Ebf* and promote a pharyngeal muscle fate in the second heart precursors, if not for the activity of Nk4/Nkx2-5, which inhibits the maintenance of *Tbx1/10* expression and directly represses Ebf activation in the SHPs [36]. The latter activities, hinting at cross-regulatory antagonistic interactions between early regulators of the heart and pharyngeal muscle programs, which contribute to their segregation to distinct precursor cells. How many of these relate to CHDs in humans? Mutations in the human homolog NKX2-5 cause various forms of viable CHDs, and Nkx2-5 mutant animals do not entirely lack a heart [92,93,94,95]. Instead, Nkx2-5 probably also functions in SHF progenitors, in part to oppose precocious differentiation [80,95] and either cooperate with or antagonize Tbx1 activities [82,96,97]. Thus, in spite of profound modifications of the complex morphogenetic processes observed between vertebrates and their closest relatives, there is an intriguing possibility for basic cellular and molecular mechanisms to be conserved deeply enough for studies using basal Chordates to illuminate the molecular etiology of congenital heart diseases, as was the case more than 20 years ago when fly genetics first identified the conserved cardiac determinant *tinman/Nkx2-5* [3,98], which later proved one of the main loci for CHD-causing mutations [92].

## 5. *Ciona* as an Experimental Model for Cardiac Regeneration

Regenerative animal models provide insights into the basic cell biology and unveil novel molecular mechanisms. Cardiac regeneration has been reported in vertebrates, such as newts [99,100], frogs [101] and, most recently, in zebrafish following multiple types of cardiac injury [102,103,104]. Elucidation of the successful models of cardiac regeneration that exist in nature can help illuminate therapeutic avenues toward inducing heart repair. The zebrafish model system has shown regenerative potential of cardiac myocytes via dedifferentiation of pre-existing cardiac myocytes with subsequent proliferation [105,106], whereas murine hearts have been shown to be capable of limited cardiac regeneration via transdifferentiation of epicardial cells into cardiac myocytes [107]. While there are distinct attributes for the use of zebrafish or mice as useful models of regeneration, here we propose *Ciona* as an additional regenerative model system that can provide further understanding of basic cardiac myocyte biology and the underlying blueprint of heart development and maturation.

Adult mammalian cardiac myocytes are traditionally thought of as, primarily, terminally-differentiated postmitotic cells. However, studies have shown that the adult human heart has a limited capacity for replacement of cardiac myocytes via mitotic events [108,109]. Nonetheless, the rate of replacement of functional cardiac myocytes is insufficient to repair the adult heart after damage, which results in high morbidity following cardiac injury. In order to fully understand the mechanism of cardiac myocyte proliferation during development and the transition to a postmitotic cell, the basic cell biology of cardiac myocytes must be closely examined and compared across species capable of regeneration and those that are not.

Studies of regeneration in *Ciona* have been conducted over the past 125 years (reviewed in [110]). Recent reports have shown that *Ciona* have a high capacity to regenerate the neural complex, the oral siphon, and associated organs [111,112]. The regeneration process, including the maintenance of proliferative activity in replacement cells, has been attributed to Notch signaling [113], which is similar to regenerative processes observed in zebrafish cardiac regeneration [114]. The relationship between Notch signaling and cardiac regeneration in *Ciona* has not been examined; however, it presents an intriguing avenue of future research. In addition, *Ciona* also presents a model system wherein the effects of aging on tissue repair and regeneration can be addressed. Regeneration in *Ciona* has been shown to be negatively impacted by age and/or size of the animal (the two being related; [23,111,112,115]). These studies suggest that the regenerative potential might be the greatest during juvenile and young adult stages and declining in adults; however, it is not yet known if an age-related decline in regenerative potential exists in the heart of *Ciona*. Nonetheless, the decline in regenerative potential with age in the organs that have been studied in *Ciona* is similar to observations in postnatal and adult cardiac myocytes in mammals. In 2009, a study demonstrated that the adult human heart is capable of replacing cardiac myocytes at a slow rate [108]. This rate of renewal in humans is about 1% at age 25 and declines with age. However, the rate at which mammalian cardiac myocytes are replaced throughout lifespan is controversial [116,117] and the mechanism of replacement is not yet known.

There are several benefits of using *Ciona* as a model system to study regeneration and rich opportunities for further study. First, there is abundant information about development of *Ciona* through the larval stages [39,118] and public databases chronicle gene expression throughout the lifespan of *Ciona* as well as other ascidian species [119,120,121]. In addition, since *Ciona* have a short life span and can be reared from egg to adult in closed marine systems [122], the entire life cycle of *Ciona* may be examined. Second, since the *Ciona* genome has been sequenced and annotated [16], *Ciona* are amenable to many sophisticated molecular tools, including transgenic lines with specifically-expressed molecular markers [123] that have been used to track regenerative responses [111]. Furthermore, most developmental control genes often reactivated in regeneration are present in *Ciona*, but in single copies, facilitating functional analyses. Individual adult organs from *Ciona* can also be cultured as explants in order to manipulate and examine responses *in vitro* [111]. Whole organs have been removed from *Ciona*, which then fully regenerated an intact organ [124]; however, this has not been repeated using modern methods. Notably, the adult *Ciona* heart can be dissected out of the body and survive in culture for up to 10 days (unpublished data, HJEA), which presents an opportunity for further study. Finally, using skeletal muscles as an analogy, detailed knowledge of the early clonal origins of cardiomyocytes opens the possibility to analyze how adult stem cells may emerge from undifferentiated progenitors in swimming larvae. Other Tunicates, such as colonial Ascidians, have the capacity to form cardiac cells as well as other somatic progenitor cells from hemoblasts, which demonstrates wide cellular potential [125]. Other invertebrate regeneration can occur via non-proliferative tissue repair wherein postmitotic cells undergo polyploidization and fusion [126]. Thus, it is evident that regenerative processes and the regulatory mechanisms that mediate the complex state of cells in adult tissue are difficult to decipher; however, the *Ciona* model system may illuminate possible pathways among Chordates.

**Figure 6:**
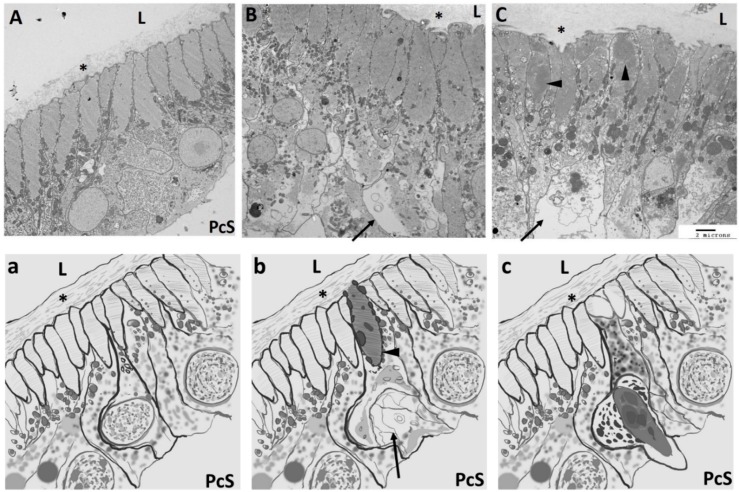
Degeneration of cardiac myocytes. Panels (**A**–**C**) are transmission electron micrographs of *Ciona* myocardium and panels (**a**–**c**) are cartoon depictions of the TEM images to highlight details and provide a proposed model for cardiac myocyte degeneration. (**A**) Normal *Ciona* myocardium displays cardiac myocytes with organized myofibrils and thin layer of extracellular matrix on the luminal side (asterisk); (**B**) 24 h post stress, myofibrils in cardiac myocytes begin to break down, spaces open toward the pericardial space (PcS, arrows), mitochondria migrate toward both poles and enlarge, and the extracellular matrix in the lumen thickens (asterisk); and (**C**) 48 h post stress, complete breakdown of the myofibrils occurs (arrow heads).

In 1953 Millar described degeneration in the myocardium of *Ciona* and growth zones of the heart [20]. The process of degeneration begins by thickening of the cardiac myocyte and breakdown of sarcomeric organization. This may be initiated in several neighboring cells. The nuclei lose their sharp outline and the sarcolemma begins to bulge into the pericardial space. The degenerated cells slough off into the pericardial space and collect in the pericardial body. The pericardiac body becomes larger as the *Ciona* ages (Figure 6). The preliminary data presented in Figure 6 closely resembles Millar’s early depiction wherein sarcomere disassembly and loss of nuclear integrity occurs in injured *Ciona* myocardium; however, the process of cardiac myocyte replacement in the *Ciona* myocardium needs further characterization (Figure 6). Notably, this process closely resembles dedifferentiation events that have been well-characterized in the zebrafish model system, which demonstrates that new cardiac myocytes come from existing cardiac myocytes during cardiac regeneration [105,106].

Cardiac myocytes in *Ciona* share many of the same structural features as mammalian cardiac myocytes, including a complex cytoskeleton with many sarcomeres and tight cell-cell junctions. However, features of mammalian cardiomyocytes such as binucleation and polyploidy might influence the ability of these cells to undergo mitotic division and may reflect the differences in their continued proliferative potential [127]. In regenerative models, such as zebrafish and newts, cardiac myocytes are predominantly mononucleated whereas in mammalian cardiac myocytes binucleation is known to increase postnatally, which coincides with decreased proliferative potential [128,129,130,131]. In addition, sarcomeric organization is much more elaborate in mammalian cardiac myocytes. Given that sarcomeres of cardiac myocytes must be disassembled in order for cell division to occur [132], it is possible that the simpler sarcomeres in *Ciona* cardiac myocytes are more amenable to break down and, thus, undergo cell division during regeneration. In the zebrafish model system, cardiac myocytes with less organized sarcomeres have higher rates of DNA synthesis and mitosis [105,106]. Furthermore, the extracellular matrix (ECM) of *Ciona* cardiac myocytes displays intermediate complexity between vertebrate ECM and matrix of non-Chordate invertebrates, such as *Drosophila* [34]. Notably, the *Ciona* ECM includes fibronectin [133], which is present only in tunicate and vertebrate genomes, and is known to be an important mediator of cardiac myocyte proliferation in mammals. In mammals, the ECM of cardiac myocytes changes significantly during the neonatal period when the proliferative potential begins to decline [134]. While further studies are required in order to understand the relationship between the regenerative potential of cardiac myocytes and ECM composition, the composition of the ECM in *Ciona* also needs to be characterized for comparison.

The *Ciona* heart has been reported to have growth zones at the inflow/outflow regions of the myocardium [9,20]. The growth zone consists of undifferentiated cells that divide rapidly to form transitional cells in which myofilaments begin to appear. Further differentiation towards mature cardiac myocytes results in sarcomeric organization of the myofilaments to form striations and elongation of the cells. The formation of new cardiac myocytes from undifferentiated precursors needs to be validated using modern methods in *Ciona*. However, this presents an additional possibility of examining transdifferentiation as a mechanism for cardiac regeneration in *Ciona*. Transdifferentiation has been identified in the zebrafish model system as a mechanism of regeneration [135,136]. Notably, transdifferentiation was not observed in the adult zebrafish heart, which could be an interesting difference between *Ciona* and zebrafish if both transdifferentiation and dedifferentiation occurs in the adult *Ciona* myocardium during regeneration. Alternatively, these “undifferentiated cells” could constitute cardiac stem cells that promote the continuous growth of the adult heart, and may also contribute the regeneration. As stated above, *Ciona* might offer a unique opportunity to study the early developmental origins of cardiac stem cells.

The regenerative potential of the *Ciona* heart has not yet been completely determined; however, the information collected here provides intriguing possibilities for further studies of the regenerative potential of the *Ciona* heart and lends the question: Are there similar mechanisms in the adult mammalian heart that can be reactivated in order to stimulate heart regeneration? The field of cardiac biology has learned much from other regenerative model systems such as the newt and the zebrafish. As a basal Chordate with conserved cardiac gene program, the *Ciona* heart serves as a living scaffold of differentiated cardiac myocytes that can help to further elucidate the blueprint of cardiac regeneration. Cardiac myocytes in *Ciona* could be pushed toward proliferation and myocardial development using conserved signaling pathway components and/or growth factors. The easily accessible *Ciona* heart could be used to study effect of blood flow on cardiac myocyte proliferation. The possibilities using modern techniques to further characterize the *Ciona* heart are vast and these studies may provide important insights into cardiac myocyte biology. 

## 6. Concluding Remarks

Vertebrate heart development is a complex process that requires the coordination of genetic programs, as well as many cellular and morphogenetic events. Studies in comparative genomics have demonstrated that the molecular determinants controlling key features in the development of Chordates are conserved. In order to fully understand how vertebrate features evolved, the genomics, development, and anatomical features of the closest invertebrate relatives provide important insights into the ancestral state of basic biological mechanisms. The *Ciona* heart displays the basal form of the Chordate heart as well as the basic cardiac genetic program and cell specification events during development and in congenital heart diseases. Moreover, the adult *Ciona* may serve as a regenerative model system to help further elucidate the differences between organisms that are capable of heart regeneration and those that are not. While the *Ciona* heart may have some divergent anatomical and histological features, the genetic underpinnings of cardiac cell specification, as well as the basic cellular structures, are deeply conserved. This level of conservation is particularly intriguing considering the regulatory states of cardiac progenitors and the conserved cardiopharyngeal origins of the first and second heart fields. Thus, as a basal Chordate, *Ciona* can provide important insights into cardiac gene regulation, as well as cardiac myocyte biology. The molecular and cellular impact of CHD-causing mutations on progenitor cells may be modeled in the simpler tunicate *Ciona* without the complexity of the redundant genome in higher vertebrates. Moreover, *Ciona* may provide an opportunity to delineate the cellular and molecular requirements for cardiac regeneration with two distinct advantages: a chance to track cellular origins back to early progenitors and the genetic and cellular simplicity. With recent advances in genetic tools (*i.e.*, CRISPR-Cas9; [137,138,139] and culturing methods [122,140] the *Ciona* model system promises to provide many more important insights into cardiac biology.

## Supplementary Materials

The following are available online at http://www.mdpi.com/2308-3425/3/3/25/s1, Video S1: Juvenile heartbeat, Video S2: Adult heartbeat.

## Abbreviations

The following abbreviations are used in this manuscript:

CHD	congenital heart defects/disease
Myo	myocardium
Pc	pericardium
PcB	pericardial body
PcS	pericardial space
L	lumen
TEM	transmission electron microscopy
ATMs	anterior tail muscle precursors
ATM	anterior tail muscles
TVCs	trunk ventral cells
FHPs	first heart precursors
STVCs	second trunk ventral cells
SHPs	second heart precursors
ASMFs	atrial siphon muscle founder cells
ASMPs	atrial siphon muscle precursors
LoM	longitudinal body wall muscles
iASMP	inner atrial siphon muscle precursor
oASMP	outer atrial siphon
OSM	oral siphon muscles
ECM	extracellular matrix
